# Supplementary material for: Scattering polarimetry enables correlative nerve fiber imaging and multimodal analysis
Source: Sci Rep. 2025 May 27;15:18493. doi: 10.1038/s41598-025-02762-w (PMC12117026; doi:10.1038/s41598-025-02762-w)
Supplement: Supplementary file 1 — Supplementary Information. [file 41598_2025_2762_MOESM1_ESM.pdf]

SUPPLEMENTARY INFORMATION:

Scattering polarimetry enables correlative nerve fiber imaging and multimodal analysis

Franca auf der Heiden[1], **Markus Axer**[1,2,*], **Katrin Amunts**[1,3], **and Miriam Menzel**[4,1,*]

[1]Institute of Neuroscience and Medicine (INM-1), Forschungszentrum Jülich GmbH, 52425 Jülich, Germany
[2]Department of Physics, School of Mathematics and Natural Sciences, University of Wuppertal, 42119 Wuppertal, Germany
[3]C. and O. Vogt Institute for Brain Research, University Hospital Düsseldorf, 40225 Düsseldorf, Germany
[4]Department of Imaging Physics, Delft University of Technology, 2628 CJ Delft, The Netherlands
[*]m.axer@fz-juelich.de, m.menzel@tudelft.nl

ABSTRACT

Mapping the intricate network of nerve fibers is crucial for understanding brain function. Three-Dimensional Polarized Light Imaging (3D-PLI) and Computational Scattered Light Imaging (ComSLI) map dense nerve fibers in brain sections with micrometer resolution using visible light. 3D-PLI reconstructs 3D-fiber orientations, while ComSLI disentangles multiple directions per pixel. So far, these imaging techniques have been realized in separate setups. A combination within a single device would facilitate faster measurements, pixelwise mapping, cross-validation of fiber orientations, and leverage the advantages of each technique while mitigating their limitations. Here, we introduce the Scattering Polarimeter, a microscope that facilitates correlative large-area scans by integrating 3D-PLI and ComSLI measurements into a single system. Based on a Mueller polarimeter, it incorporates variable retarders and a large-area light source for direct and oblique illumination, enabling combined 3D-PLI and ComSLI measurements. Applied to human and vervet monkey brain sections, the Scattering Polarimeter generates results comparable to state-of-the-art 3D-PLI and ComSLI setups and creates a multimodal fiber direction map, integrating the robust fiber orientations obtained from 3D-PLI with fiber crossings from ComSLI. Furthermore, we discuss applications of the Scattering Polarimeter for unprecedented correlative and multimodal brain imaging.

Supplementary 1: Mueller polarimetry

The Mueller polarimetry measurements with the Scattering Polarimeter were performed by setting the polarization state generator and analyzer (PSG and PSA) to all possible combinations of the basic Stokes polarization states (vertical and horizontal linear polarization, diagonal and anti-diagonal linear polarization, right-handed and left-handed circular polarization) and measuring the transmitted intensity for each configuration, resulting in 36 measurements. The required polarization settings were achieved with voltages previously determined for all four liquid crystal variable retarders (LCVRs). Subsequently, the 4×4 Mueller matrix elements were calculated by solving the resulting system of linear equations[1].

Eigenvalue calibration method

To account for non-ideal behavior of PSG and PSA, all measured Mueller matrices were calibrated following the eigenvalue calibration method (ECM) described by COMPAIN et al.[2]. The method determines the calibration matrices A and W from a Mueller polarimetry measurement without sample, a measurement with a linear polarization filter in two different orientations, and a measurement with a retarding element. An advantage of the ECM is that it does not require the azimuthal angle of the calibration samples to be precisely aligned. Here, we used a linear polarization filter (*Edmund Optics XP38*) at azimuthal angles of $\approx 0°$ and $\approx 45°$ and a quarter-wave plate (*Newport 10RP34-532*) at $\approx 45°$. From the known calibration matrices A and W, every measured Mueller matrix M_{meas} was calibrated via: $M = AM_{\text{meas}}W$.

Lu-Chipman decomposition

To derive the (linear) retardance and the fast axis (fiber) orientation from the measured Mueller matrices, the Lu-Chipman decomposition[3] was used. It rewrites the Mueller matrix M as the matrix product:

$$M = M_\Delta M_R M_D \tag{1}$$

with M_Δ the depolarization matrix, M_R the retardance matrix and M_D the diattenuation matrix. The decomposition of the retardance and the depolarization matrix includes solving an eigenvalue problem for the depolarization matrix[3].

When M is decomposed according to Eq. (1), the Mueller matrix of depolarization M_Δ yields the total depolarization Δ:

$$\Delta = 1 - \frac{|Tr(M_\Delta)|}{3}, \qquad 0 \leq \Delta \leq 1 \tag{2}$$

with $Tr(M_\Delta)$ denoting the trace of the matrix. A value of 1 indicates complete depolarization. A value of 0 indicates that no depolarization occurs.

The total retardance R can be computed from the retardance matrix M_R via:

$$R = \cos^{-1}\left(\frac{Tr(M_R)}{2} - 1\right) \qquad \text{with} \qquad M_R = \begin{pmatrix} 1 & \vec{0}^T \\ \vec{0} & m_R \end{pmatrix} \tag{3}$$

It can be separated into an optical rotation and the linear retardance δ[4,5]:

$$\delta = \cos^{-1}\left(\sqrt{\left([m_{R,11} + m_{R,22}]^2 + [m_{R,21} + m_{R,12}]^2\right)} - 1\right) \tag{4}$$

with the orientation of the fast axis with respect to the x-axis (fiber orientation) given by:

$$\phi_R = \frac{1}{2}\tan^{-1}\left(\frac{m_{R,12} - m_{R,21}}{m_{R,13} - m_{R,31}}\right). \tag{5}$$

Measured Mueller matrices of known optical elements

To test the performance of the Scattering Polarimeter as Mueller polarimeter, we performed Mueller polarimetry measurements of test samples with known optical properties and calibrated the measured Mueller matrices with the ECM as described above.

The ideal Mueller matrix for a measurement without sample $M_{\text{air, id.}}$ is the unit matrix[6]. The normalized calibrated Mueller matrix M_{air} for the empty setup was measured to be:

$$M_{\text{air}} = \begin{pmatrix} 1.0000 & 0.0009 & -0.0017 & -0.0018 \\ -0.0004 & 0.9978 & -0.0023 & -0.0004 \\ 0.0000 & 0.0027 & 0.9971 & 0.0047 \\ -0.0004 & 0.0042 & -0.0035 & 0.9967 \end{pmatrix} \qquad M_{\text{air, id.}} = \begin{pmatrix} 1 & 0 & 0 & 0 \\ 0 & 1 & 0 & 0 \\ 0 & 0 & 1 & 0 \\ 0 & 0 & 0 & 1 \end{pmatrix} \tag{6}$$

The ideal Mueller matrix for measuring a horizontal linear polarizer $M_{\text{LPH, id.}}$ contains only ones and zeros[6]. The normalized calibrated Mueller matrix M_{LPH} for a linear polarization filter (*Edmund Optics XP38*) in horizontal orientation (disregarding the pre-factor of 0.5) was measured to be:

$$M_{\text{LPH}} = \begin{pmatrix} 1.0000 & 0.9904 & 0.1171 & -0.0001 \\ 1.0349 & 0.9910 & 0.1192 & 0.0068 \\ 0.0174 & 0.0209 & -0.0059 & -0.0312 \\ 0.0032 & -0.0064 & 0.0301 & 0.0201 \end{pmatrix} \qquad M_{\text{LPH, id.}} = \begin{pmatrix} 1 & 1 & 0 & 0 \\ 1 & 1 & 0 & 0 \\ 0 & 0 & 0 & 0 \\ 0 & 0 & 0 & 0 \end{pmatrix} \tag{7}$$

The matrix element $m_{10} = 1.0349$ is larger than m_{00}. This can happen due to statistical fluctuations, but applying the criteria suggested by DEL HOYO ET AL.[7] sets the matrix element to 1 which yields a physically correct Mueller matrix M_{LPH}.

The ideal Mueller matrix of a birefringent plate $M_{\text{QWP', id.}}$ (*Newport 10RP34-532*) was modeled as a phase retarder with $\delta = 0.255$, i.e. $\cos(2 \cdot \pi \cdot 0.255) \approx -0.03$ and $\sin(2 \cdot \pi \cdot 0.255) \approx 1$, hence taking the offset from the ideal quarter-wave plate (QWP) into account by using a phase shift of 0.255 instead of 0.25[6]. The normalized calibrated Mueller matrix $M_{\text{QWP'}}$ of the birefringent plate was measured as:

$$M_{\text{QWP'}} \approx \begin{pmatrix} 1.0000 & 0.0203 & 0.0131 & 0.0222 \\ -0.0136 & -0.0518 & 0.1114 & -0.8284 \\ -0.0014 & 0.055 & 0.8889 & 0.0561 \\ 0.0138 & 0.8910 & -0.0990 & -0.0601 \end{pmatrix} \qquad M_{\text{QWP', id.}} \approx \begin{pmatrix} 1 & 0 & 0 & 0 \\ 0 & -0.03 & 0 & -1 \\ 0 & 0 & 1 & 0 \\ 0 & 1 & 0 & -0.03 \end{pmatrix} \tag{8}$$

For the empty setup, the matrix elements are in accordance with the theoretical matrix down to the third or even fourth decimal place. For the linear polarization filter, the matrix elements deviate mostly in the third or second decimal position. For the birefringent plate, the matrix elements mostly deviate in the second decimal place. Overall, the performance of the Scattering Polarimeter is comparable to other Mueller polarimeters in literature[8,9].

Supplementary 2: Signal analysis in Three-dimensional Polarized Light Imaging (3D-PLI)

For a 3D-PLI measurement, the sample is illuminated by linearly polarized light under different azimuthal rotation angles ρ. The linearly polarized light passes through the sample (thin cryo-sectioned brain tissue) and the optical anisotropy of the myelinated nerve fibers introduces a phase shift depending on the local nerve fiber orientation which changes the polarization to elliptical. To analyze the phase shift, the light passes through a circular analyzer. In the Scattering Polarimeter, polarization state generator and analyzer are realized by a set of liquid crystal variable retarders and fixed linear polarizers (see main Fig. 1a). In the state-of-the-art 3D-PLI setup that was used as reference measurement (LMP3D, see Methods), the ingoing linear polarization is generated by a rotating linear polarizer, and the circular analyzer is realized by a quarter-wave plate followed by another linear polarizer. The pixelwise evaluation of a 3D-PLI signal is depicted in main Fig. 2b. The light intensity $I(\rho)$ of every image pixel is described by a sinusoidal curve[10]:

$$I(\rho) = \frac{I_0}{2} \cdot (1 + \sin(2\rho - 2\phi) \cdot \sin(\delta)), \tag{9}$$

where δ is the retardance, $\sin(\delta)$ is the retardation, ρ is the angle of ingoing linear polarization, and ϕ is the fiber direction angle (projected onto the brain section plane with respect to the x-axis of the setup). The local fiber inclination (i.e., the out-of-plane angle) α is connected to the local retardance:

$$\delta = \frac{2\pi d}{\lambda} \Delta n \cos^2(\alpha). \tag{10}$$

The retardation $|\sin(\delta)|$ is linked to the amplitude ΔI of the intensity curve $I(\rho)$ via: $\Delta I \propto |\sin(\delta)|$. The phase of $I(\rho)$ is determined by the fiber direction (i.e. the in-plane angle) ϕ.

A discrete harmonic Fourier decomposition allows the evaluation of the curve $I(\rho)$[11]:

$$I(\rho) = \frac{I_0}{2} \cdot (1 + \sin(2\rho - 2\phi) \cdot \sin(\delta)) = a_0 + a_2 \cdot \sin(2\rho) + b_2 \cdot \cos(2\rho) \tag{11}$$

with the coefficients

$$a_0 = \frac{I_0}{2}, \qquad a_2 = \frac{I_0}{2} \cdot \sin(\delta) \cdot \cos(2\phi), \qquad b_2 = \frac{I_0}{2} \cdot \sin(\delta) \cdot \sin(2\phi). \tag{12}$$

The Fourier coefficients a_0, a_2 and b_2 are calculated for each pixel from the individual light intensity I_i for all equidistant angles ρ_i for N sampled angular steps i:

$$a_0 = \frac{1}{N} \sum_{N}^{i=1} I_i, \qquad a_2 = \frac{2}{N} \sum_{N}^{i=1} I_i \sin(2\rho_i), \qquad b_2 = \frac{2}{N} \sum_{N}^{i=1} I_i \cos(2\rho_i). \tag{13}$$

From the Fourier coefficients, the transmittance map, the retardation map, and the fiber direction map can be computed. The *transmittance map* corresponds to the average of the signal and represents the birefringence-independent light extinction of the sample. White and gray matter have distinct attenuation coefficients in the optical regime, with the attenuation through white matter being generally larger due to higher absorption and scattering. The transmittance τ is calculated pixelwise from the zeroth Fourier coefficient as:

$$I_0 = \tau = 2a_0. \tag{14}$$

The *retardation map* shows the parameter $|\sin(\delta)| = \Delta I / I_0$, i.e. the normalized amplitude of the light intensity profile. The retardation $|\sin(\delta)|$ is computed from the three Fourier coefficients:

$$|\sin(\delta)| = \frac{\sqrt{a_2^2 + b_2^2}}{a_0} \in [0, 1]. \tag{15}$$

The fiber direction map shows the azimuthal (in-plane) angle ϕ_{PLI} of each fiber given by:

$$\phi_{\text{PLI}} = 180° \cdot \frac{\arctan 2(-a_2, b_2)}{2\pi} \in [-90°, 90°] \tag{16}$$

and shifted by 90° to be in the angular range $\in [0°, 180°]$.

Supplementary 3: Fiber directions from all modalities

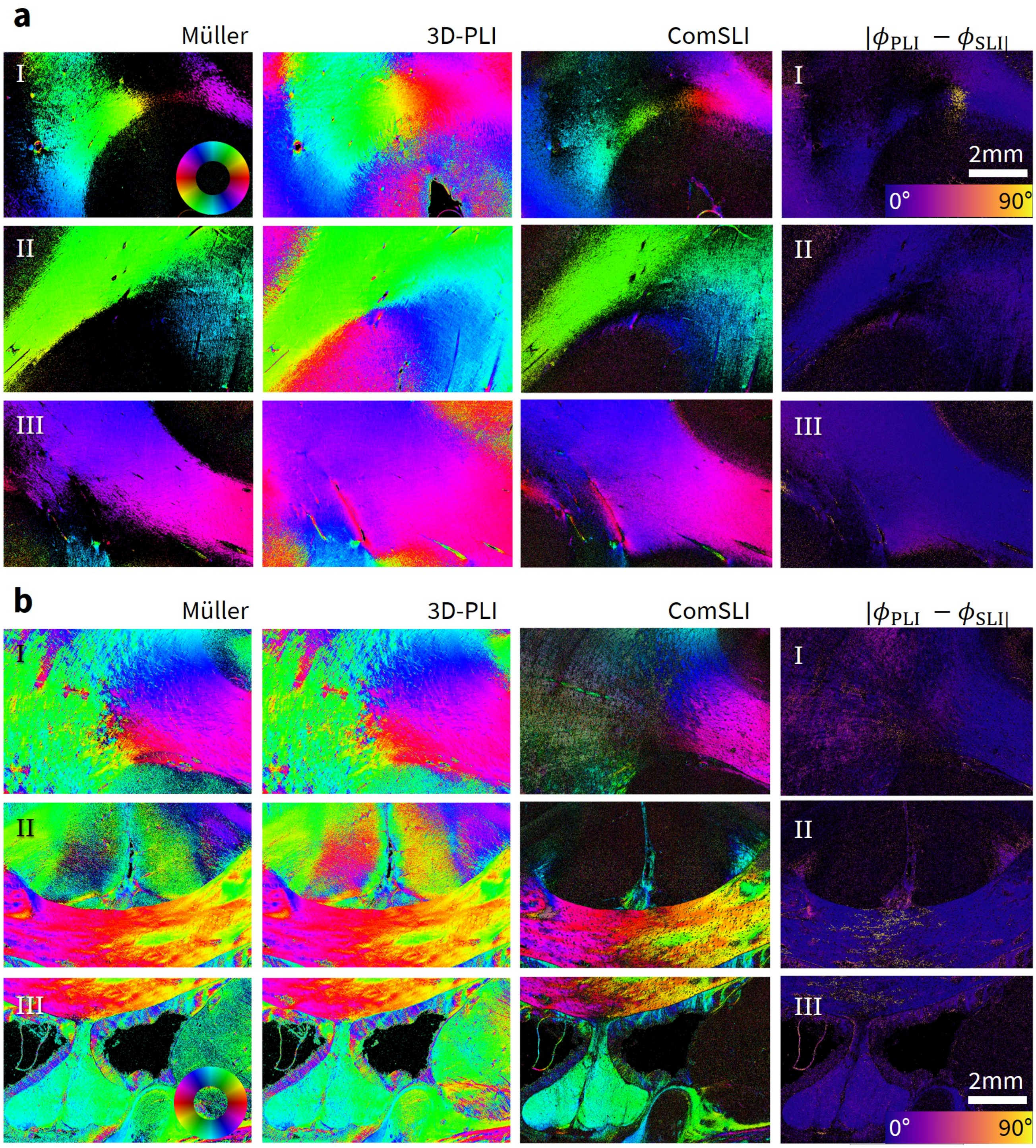

Supplementary Fig. 1. Comparison of fiber directions from all modalities: Mueller polarimetry, 3D-PLI and ComSLI (all three directions displayed in a fiber direction map). The fourth column shows the absolute difference in fiber direction between 3D-PLI and the closest direction obtained by ComSLI $|\phi_{PLI} - \phi_{SLI}|$. Background areas are displayed in black. (**a**) Human brain section (high scattering, low transmittance). (**b**) Vervet brain section (low scattering, high transmittance).

Supplementary 4: Calculation of systematic errors

To uncover statistical and systematic errors within the setup, image pixels from two characteristic regions of the human brain section (low transmittance, high scattering) and the vervet monkey brain section (high transmittance, low scattering) were investigated in detail.

The insets in Supplementary Figs. 2a and 2c show the transmittance map for the human and the vervet brain section, respectively, with two locations marked for each: one in white matter (magenta star), where a sinusoidal curve with high retardation (i.e. large amplitude) but comparatively low transmittance is expected. Reference measurements indicated parallel fibers in the chosen white matter area. The phase of the sinusoidal curve depends on the fiber direction but also on the circular analyzer setting of the PSA. The right-handed circular analyzer (RC) setting results in a 90° shift compared to the left-handed circular analyzer (LC). This shift must be accounted for in the correct mathematical evaluation of fiber directions but has no other consequences. Furthermore, a region without tissue (magenta diamond) was investigated, accordingly with high transmittance and only statistical noise, both due to the absence of birefringent tissue.

Figures 2a and 2c display the raw intensities averaged over 20×20 image pixels, thus limiting the impact of single pixel artifacts, blood vessels, or crystals within the embedding medium. The statistical error σ_I=17 a.u. was estimated from the average standard deviation of 4 raw images. The sinusoidal curve is displayed for the right-circular (RC, blue) and left-circular (LC, orange) setting. A dashed line indicates the fitted sinusoidal curve. Figures 2b and 2d show the corresponding residuals. The coefficient of determination R^2 indicates the fit quality for each image pixel:

$$R^2 = 1 - \frac{\sum(y_i - f_i)^2}{\sum(y_i - \bar{y})^2} \in [0,1] \tag{17}$$

where y_i represents the measured data points (here, intensity in arbitrary units), f_i the fitted data, and $\bar{y}$ the mean value of the measured data. The closer R^2 is to 1, the better the fit quality typically is. With a coefficient of determination R^2 close to 1, the Fourier coefficient fit can generally be considered successful, especially for white matter areas.

However, the mean amplitude (i.e. transmittance) differs slightly between RC and LC, indicating a polarization dependency of transmittance. This phenomenon is not based on any physical tissue properties as confirmed by the reference measurements but caused by minor ellipticities within the scattering polarimeter, i.e. unintended elliptical deviations from the ideal circular or linear polarization.

Furthermore, the residuals indicate a systematic error in the shape of a higher-frequency sinusoidal curve. This systematic error is relatively small compared to the maximum amplitude of the signal in white matter and even smaller for the background signal, suggesting that the higher-frequency curve either originates from the tissue itself or is enhanced through interaction with the tissue.

Mueller matrix calculus provides further insight into the observed systematic errors, as demonstrated in the following. Fibrous brain tissue with a retardance δ and a fiber direction ϕ can be modeled as a Mueller matrix of a retarder rotated around an azimuthal angle ϕ[12]:

$$M_{\text{tissue}} \tag{18}$$

$$= R(\phi) \cdot M(\delta) \cdot R(-\phi) \tag{19}$$

$$= \begin{pmatrix} 1 & 0 & 0 & 0 \\ 0 & \cos(2\phi) & -\sin(2\phi) & 0 \\ 0 & \sin(2\phi) & \cos(2\phi) & 0 \\ 0 & 0 & 0 & 1 \end{pmatrix} \begin{pmatrix} 1 & 0 & 0 & 0 \\ 0 & 1 & 0 & 0 \\ 0 & 0 & \cos\delta & \sin\delta \\ 0 & 0 & -\sin\delta & \cos\delta \end{pmatrix} \begin{pmatrix} 1 & 0 & 0 & 0 \\ 0 & \cos(2\phi) & \sin(2\phi) & 0 \\ 0 & -\sin(2\phi) & \cos(2\phi) & 0 \\ 0 & 0 & 0 & 1 \end{pmatrix} \tag{20}$$

$$= \begin{pmatrix} 1 & 0 & 0 & 0 \\ 0 & \cos^2(2\phi) + \sin^2(2\phi)\cos\delta & \cos(2\phi)\sin(2\phi)(1-\cos\delta) & -\sin(2\phi)\sin\delta \\ 0 & \cos(2\phi)\sin(2\phi)(1-\cos\delta) & \sin^2(2\phi) + \cos^2(2\phi)\cos\delta & +\cos(2\phi)\sin\delta \\ 0 & +\sin(2\phi)\sin\delta & -\cos(2\phi)\sin\delta & \cos\delta \end{pmatrix} \tag{21}$$

$$= \begin{pmatrix} 1 & 0 & 0 & 0 \\ 0 & w_\phi^2 + v_\phi^2\cos\delta & w_\phi v_\phi(1-\cos\delta) & -v_\phi\sin\delta \\ 0 & w_\phi v_\phi(1-\cos\delta) & v_\phi^2 + w_\phi^2\cos\delta & +w_\phi\sin\delta \\ 0 & +v_\phi\sin\delta & -w_\phi\sin\delta & \cos\delta \end{pmatrix} \tag{22}$$

Here, the abbreviations $v_\phi = \sin(2\phi)$, $w_\phi = \cos(2\phi)$ are used. Diattenuation and depolarization are neglected.

Diattenuation is neglected because previous studies have shown that the diattenuation of brain tissue does not have much impact on 3D-PLI signals[13].

Depolarization is neglected because depolarization values of healthy white matter are expected to be relatively low, also in formalin-fixed tissues (GROS et al.[14] reported values between 0.9–1.0). Furthermore, depolarization effects do not affect the in-plane fiber orientations from 3D-PLI or the transmittance, but only the determined retardance values, which were not analyzed quantitatively in this study. This can be seen as follows: Assuming that brain tissue also acts as a non-uniform partial depolarizer characterized by depolarization indices Δ_a, Δ_b, Δ_c, it is possible to extend the mathematical model to incorporate depolarization effects:

$$\begin{pmatrix} 1 & 0 & 0 & 0 \\ 0 & \Delta_a & 0 & 0 \\ 0 & 0 & \Delta_b & 0 \\ 0 & 0 & 0 & \Delta_c \end{pmatrix} \cdot M_{\text{tissue}} \tag{23}$$

Multiplying the resulting matrix with the matrix of a circular analyzer (from the left) and the Stokes vector of ingoing linear polarization with polarization angle ρ (from the right) yields for the intensity of a 3D-PLI measurement:

$$I = \frac{I_0}{2}\left(1 + \Delta_c \sin(2\rho - 2\phi)\sin(\delta)\right) \tag{24}$$

As can be seen, the amplitude of the signal (retardance) is affected by the depolarization index, but not the phase of the signal (fiber orientation) or the average (transmittance).

Mueller calculus for a non-ideal Mueller polarimeter

Ideal and non-ideal Mueller polarimeters can be modeled using Mueller matrix calculus. In the following, the impact of non-ideal optical properties (usually arising from the non-ideal optical components) within the polarization state generator (PSG) and/or the polarization state analyzer (PSA) in a non-ideal Mueller polarimeter is mathematically described. The Mueller polarimeter is composed of four variable retarders and two linear polarization filters, oriented in the same manner as in the scattering polarimeter.

With ideal retarders at azimuthal angles θ and the Stokes vector of horizontal linear polarization $\vec{S}_H$, the Stokes vector $\vec{S}_{\text{PSG}}$ for the ideal polarization state generator (PSG) is given by:

$$\vec{S}_{\text{PSG}} \tag{25}$$

$$= M(\delta_2, \theta = 0^\circ) \cdot M(\delta_1, \theta = 45^\circ) \cdot \vec{S}_H \tag{26}$$

$$= \begin{pmatrix} 1 & 0 & 0 & 0 \\ 0 & 1 & 0 & 0 \\ 0 & 0 & \cos\delta_2 & \sin\delta_2 \\ 0 & 0 & -\sin\delta_2 & \cos\delta_2 \end{pmatrix} \begin{pmatrix} 1 & 0 & 0 & 0 \\ 0 & \cos\delta_1 & 0 & -\sin\delta_1 \\ 0 & 0 & 1 & 0 \\ 0 & \sin\delta_1 & 0 & \cos\delta_1 \end{pmatrix} \begin{pmatrix} 1 \\ 1 \\ 0 \\ 0 \end{pmatrix} \tag{27}$$

$$= \begin{pmatrix} 1 & 0 & 0 & 0 \\ 0 & 1 & 0 & 0 \\ 0 & 0 & \cos\delta_2 & \sin\delta_2 \\ 0 & 0 & -\sin\delta_2 & \cos\delta_2 \end{pmatrix} \begin{pmatrix} 1 \\ \cos\delta_1 \\ 0 \\ \sin\delta_1 \end{pmatrix} \tag{28}$$

$$= \begin{pmatrix} 1 \\ \cos\delta_1 \\ \sin\delta_1 \sin\delta_2 \\ \sin\delta_1 \cos\delta_2 \end{pmatrix} \tag{29}$$

$$\stackrel{\delta_2 = +\frac{\pi}{2}}{=} \begin{pmatrix} 1 \\ \cos\delta_1 \\ \sin\delta_1 \\ 0 \end{pmatrix} \tag{30}$$

with δ_1 the retardance of LCVR 1 and δ_2 the retardance of LCVR 2. When LCVR 2 is set to a retardance of $\delta_2 = +\pi/2$ for 3D-PLI measurements, the generated polarization is linear and its polarization angle spins counter-clockwise with increasing δ_1 around the plane of the Poincaré sphere.

A non-ideal PSG can deviate from the ideal values for δ_1 and δ_2, usually because of a minor voltage offset for LCVR 1 and LCVR 2. While a deviation from δ_1 only results in a global phase shift, a deviation for δ_2 causes elliptical polarization that

propagates through the whole system. When $(\delta_2 - \pi/2) = h_2 \approx 0$, a Taylor approximation to the 1st order with

$$\cos(\delta_2 \approx \frac{\pi}{2}) = -(\delta_2 - \frac{\pi}{2}) + ... \approx -h_2 \tag{31}$$

$$\sin(\delta_2 \approx \frac{\pi}{2}) = 1 - ... \approx 1 \tag{32}$$

describes the Stokes vector $\vec{S}_{\text{real}}$ as a sum of the ideal vector $\vec{S}_{\text{PSG}}$ and a deviation $\vec{S}_{h2}$:

$$\vec{S}_{\text{real}} = \begin{pmatrix} 1 \\ \cos\delta_1 \\ \sin\delta_1 \sin\delta_2 \\ \sin\delta_1 \cos\delta_2 \end{pmatrix} \approx \begin{pmatrix} 1 \\ \cos\delta_1 \\ \sin\delta_1 \\ 0 \end{pmatrix} - \begin{pmatrix} 0 \\ 0 \\ 0 \\ h_2 \sin\delta_1 \end{pmatrix} = \vec{S}_{\text{PSG}} + \vec{S}_{h2} \tag{33}$$

With the matrices for an ideal retarder and horizontal linear polarization filter at azimuthal angles θ, the ideal PSA can be described by[6]:

$$M_{\text{PSA}} \tag{34}$$

$$= M_{\text{LP}}(\theta = 0°) \cdot M(\delta_4, \theta = 45°) \cdot M(\delta_3, \theta = 0°) \tag{35}$$

$$= \frac{1}{2}\begin{pmatrix} 1 & 1 & 0 & 0 \\ 1 & 1 & 0 & 0 \\ 0 & 0 & 0 & 0 \\ 0 & 0 & 0 & 0 \end{pmatrix} \begin{pmatrix} 1 & 0 & 0 & 0 \\ 0 & \cos\delta_4 & 0 & -\sin\delta_4 \\ 0 & 0 & 1 & 0 \\ 0 & \sin\delta_4 & 0 & \cos\delta_4 \end{pmatrix} \begin{pmatrix} 1 & 0 & 0 & 0 \\ 0 & 1 & 0 & 0 \\ 0 & 0 & \cos\delta_3 & \sin\delta_3 \\ 0 & 0 & -\sin\delta_3 & \cos\delta_3 \end{pmatrix} \tag{36}$$

$$= \frac{1}{2}\begin{pmatrix} 1 & \cos\delta_4 & \sin\delta_4 \sin\delta_3 & -\sin\delta_4 \cos\delta_3 \\ 1 & \cos\delta_4 & \sin\delta_4 \sin\delta_3 & -\sin\delta_4 \cos\delta_3 \\ 0 & 0 & 0 & 0 \\ 0 & 0 & 0 & 0 \end{pmatrix} \tag{37}$$

$$\overset{\substack{\delta_3=0 \\ \delta_4=\pm\frac{\pi}{2}}}{=} \frac{1}{2}\begin{pmatrix} 1 & 0 & 0 & \mp 1 \\ 1 & 0 & 0 & \mp 1 \\ 0 & 0 & 0 & 0 \\ 0 & 0 & 0 & 0 \end{pmatrix} \tag{38}$$

with $\delta_3 = 0$ and $\delta_4 = \pm\pi/2$ when analyzing either right- or left-circular polarization in 3D-PLI.

A non-ideal PSA can deviate from the ideal values for δ_3 and δ_4, usually because of a minor voltage offset for LCVR 3 and LCVR 4, respectively. When $(\delta_4 - \pi/2) = h_4$ and $(\delta_3 - \pi/2) = h_3$, a Taylor approximation to the 1st order with:

$$\cos(\delta_4 \approx \pm\frac{\pi}{2}) = \mp(\delta_4 - \frac{\pi}{2}) + ... \approx \mp h_4 \tag{39}$$

$$\sin(\delta_4 \approx \pm\frac{\pi}{2}) = \pm 1 - ... \approx \pm 1 \tag{40}$$

$$\cos(\delta_3 \approx 0) = 1 - ... \approx 1 \tag{41}$$

$$\sin(\delta_3 \approx 0) = (\delta_3 - 0) - ... \approx h_3 \tag{42}$$

describes the whole Mueller matrix for the PSA M_{real} as a sum of two matrices, one for the ideal matrix M_{PSA} and a deviation $M_{h3,h4}$:

$$M_{\text{real}} \tag{43}$$

$$= M_{\text{LP}}(\theta = 0°) \cdot M(\delta_4, \theta = 45°) \cdot M(\delta_3, \theta = 0°) \tag{44}$$

$$= \frac{1}{2}\begin{pmatrix} 1 & \cos\delta_4 & \sin\delta_4 \sin\delta_3 & -\sin\delta_4 \cos\delta_3 \\ 1 & \cos\delta_4 & \sin\delta_4 \sin\delta_3 & -\sin\delta_4 \cos\delta_3 \\ 0 & 0 & 0 & 0 \\ 0 & 0 & 0 & 0 \end{pmatrix} \tag{45}$$

$$\approx \frac{1}{2}\begin{pmatrix} 1 & 0 & 0 & \mp 1 \\ 1 & 0 & 0 & \mp 1 \\ 0 & 0 & 0 & 0 \\ 0 & 0 & 0 & 0 \end{pmatrix} + \frac{1}{2}\begin{pmatrix} 0 & \mp h_4 & \pm h_3 & 0 \\ 0 & \mp h_4 & \pm h_3 & 0 \\ 0 & 0 & 0 & 0 \\ 0 & 0 & 0 & 0 \end{pmatrix} \tag{46}$$

$$= M_{\text{PSA}} + M_{h3,h4} \tag{47}$$

It is important to note that h_4 is not necessarily equal for $\delta_4 \approx \pi/2$ and $\delta_4 \approx -\pi/2$, i.e. can be different for right- and left-circular polarization because different voltage settings are applied to LCVR 4. However, h_3 remains equal because the setting for LCVR 3 $\delta_3 \approx 0$ is kept for both cases.

Error propagation in 3D-PLI

In the following, the error propagation in 3D-PLI caused by non-ideal optical elements in a Mueller polarimeter is calculated, based on Mueller calculus and Taylor approximations of the first order. The mathematical results aid in identifying the sources of non-ideal behavior.

Here, we assume that an offset in the retardance of the LCVRs is the primary source of systematic errors. In comparison, the azimuthal angles of the LCVRs are of secondary importance. The main reason for assuming ideal angles lies in the hardware: azimuthal angles can be set with high precision using rotation mounts. In contrast, the retardance of the LCVRs must first be characterized to relate voltage settings to actual retardance values. Furthermore, the voltage–retardance curves exhibit inflection points at retardance values of $\pm\frac{\pi}{2}$ (i.e., when operated as quarter-wave plates), where the curves are particularly steep and thus highly sensitive to small errors. This increases the likelihood of deviations from the ideal retardance. By contrast, rotation mounts offer straightforward, linear, and precise adjustment of angular settings.

The Mueller matrix for brain tissue Eq. (18), the ideal Mueller matrix of the PSA M_{PSA} and the ideal Stokes vector of the PSG $\vec{S}_{\text{PSG}}$ can be applied to calculate the Stokes vector $\vec{S}_{\text{PLI}}$ for 3D-PLI which directly corresponds to the measured intensity.

In 3D-PLI, δ_1 is variable, $\delta_2 = \pi/2$, $\delta_3 = 0$ and $\delta_4 = \pm\pi/2$:

$$\vec{S}_{\text{PLI}} \tag{48}$$

$$= M_{\text{PSA}} \cdot M_{\text{tissue}} \cdot \vec{S}_{\text{PSG}} \tag{49}$$

$$= M_{\text{PSA}} \begin{pmatrix} 1 & 0 & 0 & 0 \\ 0 & \cos^2(2\phi)+\sin^2(2\phi)\cos\delta & \cos(2\phi)\sin(2\phi)(1-\cos\delta) & -\sin(2\phi)\sin\delta \\ 0 & \cos(2\phi)\sin(2\phi)(1-\cos\delta) & \sin^2(2\phi)+\cos^2(2\phi)\cos\delta & +\cos(2\phi)\sin\delta \\ 0 & +\sin(2\phi)\sin\delta & -\cos(2\phi)\sin\delta & \cos\delta \end{pmatrix} \begin{pmatrix} 1 \\ \cos(\delta_1) \\ \sin(\delta_1) \\ 0 \end{pmatrix} \tag{50}$$

$$= \frac{1}{2} \begin{pmatrix} 1 & 0 & 0 & \mp 1 \\ 1 & 0 & 0 & \mp 1 \\ 0 & 0 & 0 & 0 \\ 0 & 0 & 0 & 0 \end{pmatrix} \begin{pmatrix} 1 \\ \cos\delta_1\cos^2(2\phi)+\sin^2(2\phi)\cos\delta+\cos(2\phi)\sin(2\phi)i(1-\cos\delta)\sin\delta_1 \\ \cos(2\phi)\sin(2\phi)(1-\cos\delta)\cos\delta_1+\sin^2(2\phi)+\cos^2(2\phi)\cos\delta\sin\delta_1 \\ +\sin(2\phi)\sin\delta\cos\delta_1-\cos(2\phi)\sin\delta\sin\delta_1 \end{pmatrix} \tag{51}$$

$$= \frac{1}{2} \begin{pmatrix} 1\pm\sin\delta\sin(\delta_1-2\phi) \\ 1\pm\sin\delta\sin(\delta_1-2\phi) \\ 0 \\ 0 \end{pmatrix} \tag{52}$$

The first element of the Stokes vector $\vec{S}_{\text{PLI}}$ is directly equivalent to the 3D-PLI signal but with $\delta_1 \hat{=} 2\rho$

$$I_{\text{PLI}} = \frac{1}{2}\left(1\pm\sin\delta\sin(\delta_1-2\phi)\right) \tag{53}$$

With the Taylor approximation in Eq. (33) and the abbreviations $v_\phi = \sin(2\phi)$, $w_\phi = \cos(2\phi)$, the non-ideal Stokes vector $\vec{S}_{\text{PLI'}}$ for a non-ideal PSG and an ideal PSA can be calculated as:

$$\vec{S}_{\text{PLI'}} \tag{54}$$

$$= M_{\text{PSA}} \cdot M_{\text{tissue}} \cdot (\vec{S}_{PSG} + \vec{S}_{h2}) \tag{55}$$

$$= \vec{S}_{\text{PLI}} + M_{\text{PSA}} M_{\text{tissue}} \vec{S}_{h_2} \tag{56}$$

$$= \vec{S}_{\text{PLI}} - \frac{1}{2} \begin{pmatrix} 1 & 0 & 0 & \mp 1 \\ 1 & 0 & 0 & \mp 1 \\ 0 & 0 & 0 & 0 \\ 0 & 0 & 0 & 0 \end{pmatrix} \begin{pmatrix} 1 & 0 & 0 & 0 \\ 0 & w_\phi^2+v_\phi^2\cos\delta & w_\phi v_\phi(1-\cos\delta) & -v_\phi\sin\delta \\ 0 & w_\phi v_\phi(1-\cos\delta) & v_\phi^2+w_\phi^2\cos\delta & +w_\phi\sin\delta \\ 0 & v_\phi\sin\delta & -w_\phi\sin\delta & \cos\delta \end{pmatrix} \begin{pmatrix} 0 \\ 0 \\ 0 \\ h_2\sin(\delta_1) \end{pmatrix} \tag{57}$$

$$= \frac{1}{2}\left[\begin{pmatrix} 1\pm\sin\delta\sin(\delta_1-2\phi) \\ 1\pm\sin\delta\sin(\delta_1-2\phi) \\ 0 \\ 0 \end{pmatrix} \pm \begin{pmatrix} h_2\cos\delta\sin\delta_1 \\ h_2\cos\delta\sin\delta_1 \\ 0 \\ 0 \end{pmatrix} \right] \tag{58}$$

$$= \vec{S}_{\text{PLI}} + \vec{S}_{\text{LCVR2}}(\delta, \delta_1) \tag{59}$$

The ideal 3D-PLI intensity curve in Eq. (53) is now modulated by a function with an amplitude h_2 that depends on the fiber retardance δ, the retardance δ_1 of LCVR 1 but not the fiber direction ϕ:

$$I_{\text{PLI'}} = \frac{1}{2}\left(1 \pm \sin\delta \sin(\delta_1 - 2\phi)\right) \pm \frac{h_2}{2}\cos\delta\sin(\delta_1). \tag{60}$$

Using the Taylor approximation in Eq. (43) and the abbreviations $v_\phi = \sin(2\phi)$, $w_\phi = \cos(2\phi)$, the non-ideal Stokes vector $\vec{S}_{\text{PLI'}}$ for an ideal PSG and a non-ideal PSA can be calculated as:

$$\vec{S}_{\text{PLI'}} \tag{61}$$

$$= (M_{\text{PSA}} + M_{h3,h4}) \cdot M_{\text{tissue}} \cdot \vec{S}_{\text{PSG}} \tag{62}$$

$$= \vec{S}_{\text{PLI}} + M_{h3,h4} \cdot M_{\text{tissue}} \cdot \vec{S}_{\text{PSG}} \tag{63}$$

$$= \vec{S}_{\text{PLI}} + \frac{1}{2}\begin{pmatrix} 0 & \mp h_4 & \pm h_3 & 0 \\ 0 & \mp h_4 & \pm h_3 & 0 \\ 0 & 0 & 0 & 0 \\ 0 & 0 & 0 & 0 \end{pmatrix} \begin{pmatrix} 1 \\ \cos\delta_1 w_\phi^2 + v_\phi^2 \cos\delta + w_\phi v_\phi (1-\cos\delta)\sin\delta_1 \\ w_\phi v_\phi (1-\cos\delta)\cos(\delta_1) + v_\phi^2 + w_\phi^2 \cos\delta \sin\delta_1 \\ v_\phi \sin\delta\cos\delta_1 - w_\phi \sin\delta\sin\delta_1 \end{pmatrix} \tag{64}$$

$$= \vec{S}_{\text{PLI}} \mp \frac{h_4}{2}\begin{pmatrix} \cos\delta_1 w_\phi^2 + v_\phi^2 \cos\delta + w_\phi v_\phi (1-\cos\delta)\sin\delta_1 \\ \cos\delta_1 w_\phi^2 + v_\phi^2 \cos\delta + w_\phi v_\phi (1-\cos\delta)\sin\delta_1 \\ 0 \\ 0 \end{pmatrix} \tag{65}$$

$$\pm \frac{h_3}{2}\begin{pmatrix} w_\phi v_\phi (1-\cos\delta)\cos\delta_1 + v_\phi^2 + w_\phi^2 \cos\delta\sin\delta_1 \\ w_\phi v_\phi (1-\cos\delta)\cos\delta_1 + v_\phi^2 + w_\phi^2 \cos\delta\sin\delta_1 \\ 0 \\ 0 \end{pmatrix} \tag{66}$$

$$= \vec{S}_{\text{PLI}} + \vec{S}_{\text{LCVR3}}(\phi,\delta,\delta_1) + \vec{S}_{\text{LCVR4}}(\phi,\delta,\delta_1) \tag{67}$$

The ideal 3D-PLI intensity curve in Eq. (53) is now modulated by two functions with amplitudes h_3 and h_4 that depend on the fiber retardance δ, the retardance of LCVR 1 δ_1, and fiber direction ϕ:

$$I_{\text{PLI'}} = \frac{1}{2}\left(1 \pm \sin\delta\sin(\delta_1 - 2\phi)\right) \tag{68}$$

$$\mp \frac{h_4}{2}\left(\cos\delta_1 w_\phi^2 + v_\phi^2\cos\delta + w_\phi v_\phi (1-\cos\delta)\sin\delta_1\right) \tag{69}$$

$$\pm \frac{h_3}{2}\left(w_\phi v_\phi (1-\cos\delta)\cos\delta_1 + v_\phi^2 + w_\phi^2\cos\delta\sin\delta_1\right) \tag{70}$$

With $v_\phi^2 = \sin^2(2\phi) = \frac{1}{2}(1-\cos 4\phi)$ and $w_\phi^2 = \cos^2(2\phi) = \frac{1}{2}(1+\cos 4\phi)$, a higher-order frequency is introduced into the signal that depends on the fiber direction.

The transmitted Stokes vector $\vec{S}_{\text{PSA'}}$ for a non-ideal PSG and a non-ideal PSA can be calculated:

$$\vec{S}_{\text{out}} = (M_{\text{PSA}} + M_{h3,h4}) \cdot M_{\text{tissue}} \cdot (\vec{S}_{\text{PSG}} + \vec{S}_{h2}) \tag{71}$$

$$= \vec{S}_{\text{PLI}} + \vec{S}_{\text{LCVR2}} + \vec{S}_{\text{LCVR3}} + \vec{S}_{\text{LCVR4}} + M_{\text{h3, h4}} \cdot M_{\text{tissue}} \cdot \vec{S}_{\text{h2}} \tag{72}$$

which is the sum of previous results and a higher-order term. However, the multiplication of the very small factors h_2, h_3 and h_4 can be neglected.

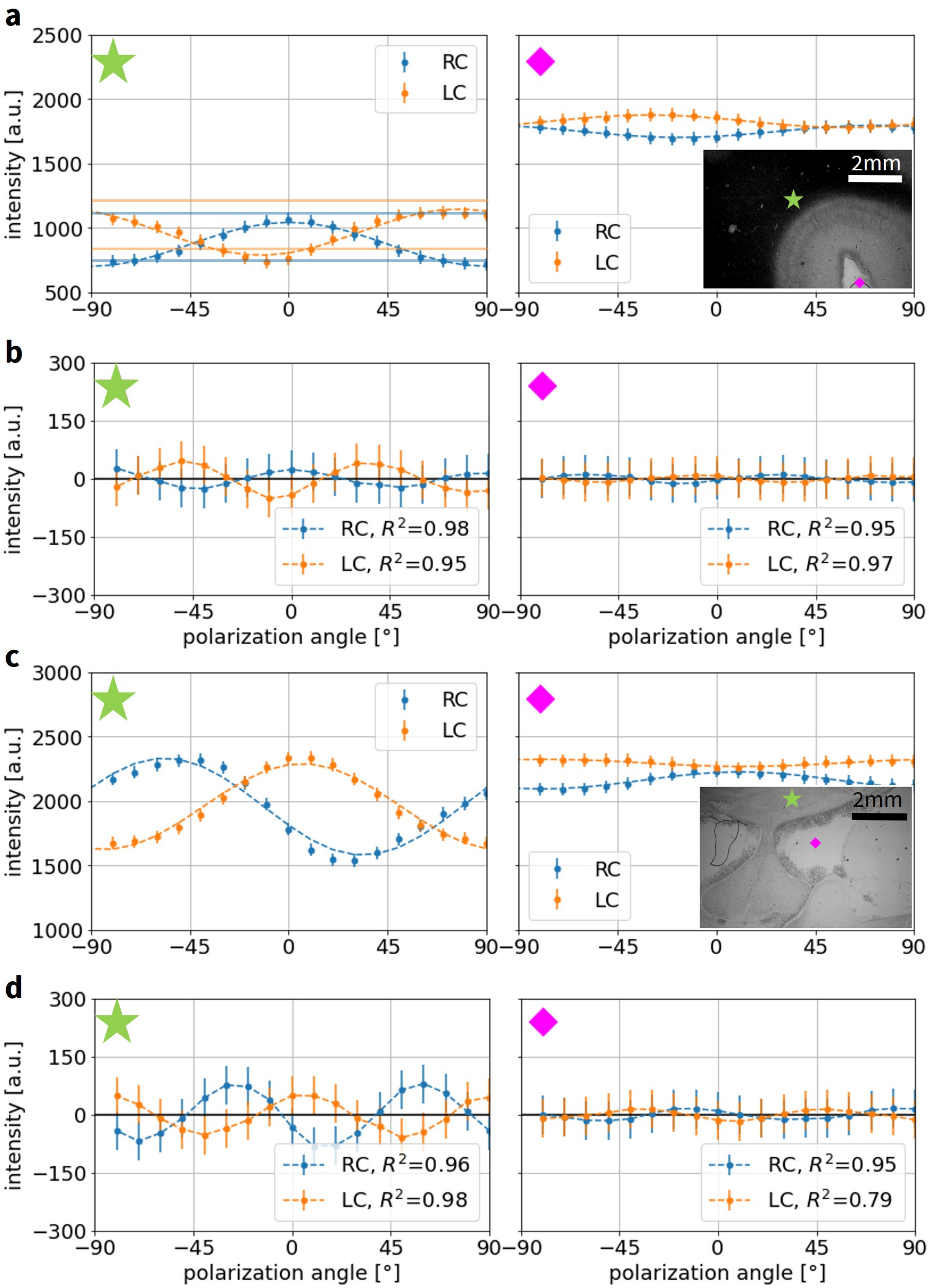

Supplementary Fig. 2. Fourier fit and residuals for exemplary pixels. 3D-PLI measurements with a left-handed circular (LC) and right-handed circular (RC) setting of the PSA are compared. (**a**) Fourier fit for the human brain section (low transmittance, high scattering). The two exemplary locations are marked in the transmittance map. (**b**) Corresponding residuals and R^2. (**c**) Fourier fit for the vervet monkey brain section (high transmittance, low scattering). The two exemplary locations are marked in the transmittance map. (**d**) Corresponding residuals and R^2 values.

References

1. Ghassemi, P. & Miranbaygi, M. H. Towards skin polarization characterization using polarimetric technique. *J. Zhejiang Univ. SCIENCE B* **10**, 602–608, DOI: https://doi.org/10.1631/jzus.b0920068 (2009).
2. Compain, E., Poirier, S. & Drevillon, B. General and self-consistent method for the calibration of polarization modulators, polarimeters, and Mueller-matrix ellipsometers. **38**, 3490–3502, DOI: https://doi.org/10.1364/ao.38.003490 (1999).
3. Lu, S.-Y. & Chipman, R. A. Interpretation of Mueller matrices based on polar decomposition. *J. Opt. Soc. Am. A* **13**, 1106, DOI: https://doi.org/10.1364/josaa.13.001106 (1996).
4. Manhas, S. *et al.* Mueller matrix approach for determination of optical rotation in chiral turbid media in backscattering geometry. *Opt. Express* **14**, 190, DOI: https://doi.org/10.1364/opex.14.000190 (2006).
5. Ghosh, N., Wood, M. F. G. & Vitkin, I. A. Mueller matrix decomposition for extraction of individual polarization parameters from complex turbid media exhibiting multiple scattering, optical activity, and linear birefringence. *J. Biomed. Opt.* **13**, 044036, DOI: https://doi.org/10.1117/1.2960934 (2008).
6. Goldstein, D. H. *Polarized light* (CRC Press, Boca Raton, Fla., 2011), third edition edn. Includes bibliographical references and index.
7. Hoyo, J. d., Sanchez-Brea, L. M. & Gomez-Pedrero, J. A. High precision calibration method for a four-axis Mueller matrix polarimeter. *Opt. Lasers Eng.* **132**, 106112, DOI: https://doi.org/10.1016/j.optlaseng.2020.106112 (2020).
8. Bueno, J. M. Polarimetry using liquid-crystal variable retarders: theory and calibration. *J. Opt. A: Pure Appl. Opt.* **2**, 216–222, DOI: https://doi.org/10.1088/1464-4258/2/3/308 (2000).
9. Baba, J. S., Chung, J.-R., DeLaughter, A. H., Cameron, B. D. & Coté, G. L. Development and calibration of an automated Mueller matrix polarization imaging system. *J. Biomed. Opt.* **7**, 341, DOI: https://doi.org/10.1117/1.1486248 (2002).
10. Axer, M. *et al.* A novel approach to the human connectome: Ultra-high resolution mapping of fiber tracts in the brain. *NeuroImage* **54**, 1091–1101, DOI: https://doi.org/10.1016/j.neuroimage.2010.08.075 (2011).
11. Glazer, A. M., Lewis, J. G. & Kaminsky, W. An automatic optical imaging system for birefringent media. *Proc. Royal Soc. London. Ser. A: Math. Phys. Eng. Sci.* **452**, 2751–2765, DOI: https://doi.org/10.1098/rspa.1996.0145 (1996).
12. Zhang, J., Guo, S., Jung, W., Nelson, J. & Chen, Z. Determination of birefringence and absolute optic axis orientation using polarization-sensitive optical coherence tomography with pm fibers. *Opt. Express* **11**, 3262, DOI: https://doi.org/10.1364/oe.11.003262 (2003).
13. Menzel, M. *et al.* Diattenuation of brain tissue and its impact on 3D polarized light imaging. *Biomed. Opt. Express* **8**, 3163, DOI: https://doi.org/10.1364/boe.8.003163 (2017).
14. Gros, R. *et al.* Effects of formalin fixation on polarimetric properties of brain tissue: fresh or fixed? *Neurophotonics* **10**, 025009, DOI: https://doi.org/10.1117/1.NPh.10.2.025009 (2023).
